# Genetic and molecular mechanism for distinct clinical phenotypes conveyed by allelic truncating mutations implicated in *FBN1*

**DOI:** 10.1002/mgg3.1023

**Published:** 2019-11-27

**Authors:** Mao Lin, Zhenlei Liu, Gang Liu, Sen Zhao, Chao Li, Weisheng Chen, Zeynep Coban Akdemir, Jiachen Lin, Xiaofei Song, Shengru Wang, Qiming Xu, Yanxue Zhao, Lianlei Wang, Yuanqiang Zhang, Zihui Yan, Sen Liu, Jiaqi Liu, Yixin Chen, Yuzhi Zuo, Xu Yang, Tianshu Sun, Xin‐Zhuang Yang, Yuchen Niu, Xiaoxin Li, Wesley You, Bintao Qiu, Chen Ding, Pengfei Liu, Shuyang Zhang, Claudia M. B. Carvalho, Jennifer E. Posey, Guixing Qiu, Deciphering Disorders Involving Scoliosis and COmorbidities (DISCO) study, James R. Lupski, Zhihong Wu, Jianguo Zhang, Nan Wu

**Affiliations:** ^1^ Department of Orthopedic Surgery Peking Union Medical College Hospital Peking Union Medical College and Chinese Academy of Medical Sciences Beijing China; ^2^ Graduate School of Peking Union Medical College Chinese Academy of Medical Sciences Beijing China; ^3^ Beijing Key Laboratory for Genetic Research of Skeletal Deformity Beijing China; ^4^ Department of Neurosurgery Xuanwu Hospital Capital Medical University Beijing China; ^5^ College of Life and Health Sciences Northeastern University ShenYang China; ^6^ Department of Molecular and Human Genetics Baylor College of Medicine Houston TX USA; ^7^ Department of Breast Surgical Oncology National Cancer Center /National Clinical Research Center for Cancer/Cancer Hospital Chinese Academy of Medical Sciences and Peking Union Medical College Beijing China; ^8^ Department of Cardiovascular Surgery Fuwai Hospital National Center for Cardiovascular Diseases Chinese Academy of Medical Sciences and Peking Union Medical College Beijing China; ^9^ Medical Research Center & Department of Central Laboratory Peking Union Medical College Hospital Peking Union Medical College and Chinese Academy of Medical Sciences Beijing China; ^10^ Amherst College Amherst MA USA; ^11^ Baylor Genetics Laboratory Houston TX USA; ^12^ Department of Cardiology Peking Union Medical College Hospital Peking Union Medical College and Chinese Academy of Medical Sciences Beijing China; ^13^ Medical Research Center of Orthopedics Chinese Academy of Medical Sciences Beijing China; ^14^ Departments of Pediatrics Baylor College of Medicine Houston TX USA; ^15^ Texas Children's Hospital Houston TX USA; ^16^ Human Genome Sequencing Center Baylor College of Medicine Houston TX USA; ^17^ Department of Molecular and Human Genetics Baylor College of Medicine Houston TX USA

**Keywords:** dominant‐negative mechanism, *FBN1*, Marfan syndrome, Marfanoid–progeroid–lipodystrophy syndrome, targeted next generation sequencing

## Abstract

**Background:**

The molecular and genetic mechanisms by which different single nucleotide variant alleles in specific genes, or at the same genetic locus, cause distinct disease phenotypes often remain unclear. Allelic truncating mutations of *FBN1* could cause either classical Marfan syndrome (MFS) or a more complicated phenotype associated with Marfanoid–progeroid–lipodystrophy syndrome (MPLS).

**Methods:**

We investigated a small cohort, encompassing two classical MFS and one MPLS subjects from China, whose clinical presentation included scoliosis potentially requiring surgical intervention. Targeted next generation sequencing was performed on all the participants. We analyzed the molecular diagnosis, clinical features, and the potential molecular mechanism involved in the MPLS subject in our cohort.

**Results:**

We report a novel de novo *FBN1* mutation for the first Chinese subject with MPLS, a more complicated fibrillinopathy, and two subjects with more classical MFS. We further predict that the MPLS truncating mutation, and others previously reported, is prone to escape the nonsense‐mediated decay (NMD), while MFS mutations are predicted to be subjected to NMD. Also, the MPLS mutation occurs within the glucogenic hormone asprosin domain of FBN1. In vitro experiments showed that the single MPLS mutation p.Glu2759Cysfs*9 appears to perturb proper FBN1 protein aggregation as compared with the classical MFS mutation p.Tyr2596Thrfs*86. Both mutations appear to upregulate SMAD2 phosphorylation in vitro.

**Conclusion:**

We provide direct evidence that a dominant‐negative interaction of FBN1 potentially explains the complex MPLS phenotypes through genetic and functional analysis. Our study expands the mutation spectrum of *FBN1* and highlights the potential molecular mechanism for MPLS.

## Graphical abstract

In the present report, we provide genetic and functional evidence for potential dominant‐negative alleles in MPLS patients caused by truncating FBN1 variants escaping NMD. These genetic and functional investigations also include description of the first patient with MPLS of Chinese ancestry.

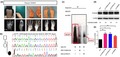

## 1 INTRODUCTION

Marfan syndrome (MFS; MIM #154700) refers to a heritable autosomal dominant disease trait of fibrous connective tissue due to heterozygous mutations in the fibrillin‐1 gene (*FBN1*; MIM #134797) on chromosome 15q21. The cardinal phenotypic features allowing for clinical diagnosis primarily occur in the skeletal, ocular, and cardiovascular systems (Dietz, 2015). MFS is manifested by clinical features/findings involving the skeletal (tall stature, disproportionately long limbs and digits [arachnodactyly], anterior chest wall deformity, mild to moderate joint laxity, and frequent spinal deformity [especially a scoliosis], cardiovascular (increased risk for aortic root dilation and/or dissection), and ocular (*ectopia lentis*) systems (Loeys et al., 2010). Marfanoid–progeroid–lipodystrophy syndrome (MPLS; MIM #616914) is a recently defined fibrillinopathy, and also a complex disease characterized by accelerated aging and postnatal lipodystrophy, poor postnatal weight gain, and characteristic dysmorphic facial features that have very rarely been clinically recognized and reported (Goldblatt, Hyatt, Edwards, & Walpole, 2011; Passarge, Robinson, & Graul‐Neumann, 2016). Recent studies have implicated a potential hormone, named asprosin, encoded by the *FBN1* locus as a mediator of the lipodystrophy phenotype (Duerrschmid et al., 2017; Romere et al., 2016). All previously reported MPLS individuals consistently harbor heterozygous truncating mutations in exon 64, which leads to the formation of premature stop codons in the C‐terminal domain of *FBN1* (Garg & Xing, 2014; Goldblatt et al., 2011; Jacquinet et al., 2014; Passarge et al., 2016; Romere et al., 2016; Song, Kim, Yoo, & Kim, 2012).

In this study, we present clinical and genetic, genomic and molecular data from three unrelated subjects with Marfan/Marfanoid syndrome, including the first case of clinically recognized MPLS in the Chinese population. The potential effects of truncating mutations of *FBN1* which are implicated in MPLS subjects conveying distinct dominant‐negative alleles and clinically distinguishable autosomal dominant (AD) disease traits were ascertained with functional assays.

## 2 MATERIALS AND METHODS

### 2.1 Ethical compliance

The study was approved by the Ethics Committee of Peking Union Medical College Hospital (PUMCH, Approval No. JS‐908). Written informed consent was provided by patients admitted into PUMCH or their corresponding guardians for the Deciphering Disorders Involving Scoliosis & Comorbidities (DISCO; http://www.discostudy.org/) study.

### 2.2 Participant ascertainment and Sample preparation

Three Marfan syndrome/Marfanoid subjects (including two proband‐only singletons and 1 parent–offspring trio family) with targeted next generation sequencing (NGS) data were extracted from the DISCO study. In brief, peripheral blood samples were collected from the affected probands and corresponding unaffected parents if available. Genomic DNA was extracted with the QIAamp DNA Blood Mini Kit (QIAGEN, Germany) according to the manufacturer's instructions.

### 2.3 Targeted next generation sequencing and Sanger sequencing

To establish an NGS panel maximally covering known genes implicated in the cause of Mendelian diseases with vertebral malformation phenotypes, we performed a systematic literature and database search for congenital scoliosis (CS) related disease. After manual review, a total of 344 genes were found, corresponding to 457 CS‐related monogenic phenotypes. Also included was an additional set of 220 genes whose protein products are involved in the pathways related to vertebral development or in which pathogenic variant alleles have been reported in association with CS phenotypes in animal models, but not yet linked to human diseases with CS phenotypes. The target panel set comprises 564 genes, 6,458 exons, and flanking 30 bp regions, and 2.97 Mb in total genomic size. DNA probes for target capture were designed and purchased from NimbleGen online (https://design.nimblegen.com/nimbledesign/app). The final set of DNA probes was expected to cover 99.7% of the targeted regions. In addition to the previously published *TBX6* featuring a compound heterozygous inheritance model (Liu et al., 2019; Wu et al., 2015; Yang et al., 2019), *FBN1* was also targeted in the panel. A targeted sequence enrichment library was prepared following a previously described protocol (Asan et al., 2011). For each sample, 1 μg of genomic DNA was used as starting material. Genomic DNA was fragmented to a size of ~200–250 bp. The fragmented DNA was end‐repaired, capped with A‐tailing and subject to ligation of indexed adaptors. After five cycles of polymerase chain reaction (PCR) amplification, each indexed product was pooled and hybridized with DNA probes for targeted capture in one solution capture reaction. The product of targeted enrichment DNA was further subject to 14 cycles of PCR amplification, followed by final library yield validation by Bioanalyzer analysis (Agilent) and quantitative polymerase chain reaction (qPCR) quantification. Sequencing was performed with the PE100 mode on an Illumina Hiseq 2500 sequencer.

After generating raw sequence data, a Perl script was used to remove low‐quality and adaptor‐contaminated reads. The remaining reads were mapped onto human reference genome assembly hg19 (GRCh37, http://genome.ucsc.edu/) with the BWA mapper. Additionally, single nucleotide variants (SNVs) and small insertions and deletions (indels) were called with the GATK (v2.2–3) bioinformatics package. Variants were annotated using an in‐house developed annotation pipeline. We filtered out common variants with high allele frequencies (minor allele frequency > 1%) in the public databases (the 1,000 Genomes Project [https://www.internationalgenome.org], the Exome Sequencing Project [http://evs.gs.washington.edu/EVS/], ExAC [http://exac.broadinstitute.org], gnomAD [https://gnomad.broadinstitute.org]) or an in‐house control dataset encompassing exome sequencing data of 2,103 subjects. We subsequently excluded silent variants which were not functionally relevant (deep intronic (>30 bp), untranslated regions (UTR), or synonymous SNVs, or noncoding indels). We also annotated the detected variants using a customized database based on the Human Gene Mutation Database (HGMD; http://www.hgmd.cf.ac.uk/) and Online Mendelian Inheritance in Man (OMIM; https://omim.org/).

Genomic DNA from all individuals was subjected to Sanger sequencing for orthogonal confirmation of all identified variants. The RefSeq accession numbers of the transcript and corresponding protein isoform of *FBN1* we used for mutation nomenclature were NM_000138.4 and NP_000129.3, respectively.

### 2.4 Prediction of nonsense‐mediated decay (NMD) events

For allelic truncating frameshift variants identified in our cohort and previously reported, in silico predictions of escaping NMD events were conducted using the online tool, NMDEscPredictor (https://nmdpredictions.shinyapps.io/shiny/) (Coban‐Akdemir et al., 2018).

### 2.5 Plasmid construction and mutagenesis

A cDNA fragment containing full‐length *FBN1* (GenBank ID: NM_000138.4) derived from human muscle cDNA and suitable restriction sites was PCR‐amplified using KOD‐Plus‐Neo (Toyobo). The PCR amplicons were cloned into the *NheI* and *SacII* sites of the pEGFP‐N1 expression vector (Clontech, Takara Bio). Mutant EGFP‐FBN1 plasmids were generated by site‐directed mutagenesis and their construction is confirmed by direct Sanger dideoxy sequencing. Gly2003Arg (G2003R) which was previously reported in an adolescent idiopathic scoliosis (AIS) case was used as a positive control (Buchan et al., 2014). Mutations of p.Tyr2596Thrfs*86 (Y2596Tfs*86), p.Glu2759Cysfs*9 (E2759Cfs*9) and Gly2003Arg were introduced into a wild‐type (WT) pEGFP‐FBN1 QuikChange Lightning Site‐directed Mutagenesis Kit (Agilent Technologies) according to the manufacturer's instructions. The resulting three mutant plasmids pEGFP‐FBN1‐Tyr2596Thrfs*86, pEGFP‐FBN1‐Glu2759Cysfs*9, and pEGFP‐FBN1‐Gly2003Arg were used for functional studies.

### 2.6 Cell culture and transfection

HEK293T cells were cultured in DMEM (Gibco) supplemented with 10% FCS (Biological Industries) and 1% penicillin/streptomycin (Gibco‐Life Technologies) at 37°C with 5% CO_2_. Cells were transfected/co‐transfected with a mutant and/or a WT pEGFP‐FBN1 using Lipofectamine 3000 (Invitrogen) according to the manufacturers’ instructions. Six hours after transfection, the medium was replaced with fresh complete DMEM, and the cells were further incubated for 48 hr. Total proteins were extracted and analyzed by western blot and SDD‐AGE, respectively.

### 2.7 Western blot

Cells were lysed with modified RIPA (50 mM Tris‐HCL, 1% NP40, 0.25% Na‐deoxycholate, 150 mM NaCl, and 1 mM EDTA; Complete^TM^ Protease Inhibitor Cocktail [Roche]), and protein concentrations were determined with the BCA‐Kit (Pierce). A total amount of 5 mg protein was size separated on an 8% SDS polyacrylamide gel, and proteins were electrophoresed and transferred to nitrocellulose membranes. Membranes were blocked in powdered milk for 30 min at room temperature (RT, 25°C), and primary antibodies (Phospho‐Smad2 (Ser245/250/255) Antibody, Cell Signaling Technology; anti‐GAPDH, Millipore) were incubated overnight at 4°C. After washing, the corresponding horseradish peroxidase–coupled goat anti‐rabbit secondary antibodies (KPL) were incubated for 1 hr at RT. Bands were visualized with the WesternBright ECL chemiluminescence system (Advansta). This experiment was performed three times with different cell lysates. Chemiluminescent signals were quantified using ImageJ (Schneider, Rasband, & Eliceiri, 2012).

### 2.8 Semi‐denaturing Detergent Agarose Gel Electrophoresis (SDD‐AGE)

For SDD‐AGE (Berchowitz et al., 2015), HEK293T cells were co‐transfected with wild type (pEGFP‐FBN1) and each mutant (pEGFP‐FBN1‐Tyr2596Thrfs*86; pEGFP‐FBN1‐Glu2759Cysfs*9) plasmid for 6 hr, and then incubated for 48 hr. After 48 hr incubation at 37°C with 5% CO_2_ in the thermotank, cells were harvested by centrifugation at 3,000 rcf for 2 min, resuspension in 200 μL water, and subsequent centrifugation. Approximately 100 μl of acid‐washed cells were then added to each well followed by 120 μL lysis buffer (100 mM Tris pH 8, 1% Triton X‐100, 50 mM β‐mercaptoethanol, 3% HALT protease inhibitor cocktail, 30 mM N‐ethylmaleimide, and 12.5 U/mL Benzonase nuclease). Blocks were then sealed with a rubber mat (Nunc 276,002) and shaken at max speed two times for 3 min on a Qiagen Tissuelyzer 2. To each well was then added 35 μL 4X sample buffer (2X TAE, 20% glycerol, 8% SDS, 0.01% bromophenol blue). The blocks were then vortexed briefly and allowed to incubate at RT for 3 min, followed by centrifugation for 2 min at 3,000 rcf to remove cell debris. Electrophoresis and capillary blotting to Hybond ECL nitrocellulose were performed as described. Proteins were transferred to a polyvinylidene difluoride membrane and probed with well‐characterized monoclonal antibody of anti‐Fibrillin 1 (Abcam ab124334).

### 2.9 Statistical analysis

SPSS software, version 17.0 (IBM Corporation), was used to conduct the two‐tailed Student's *t*‐test to compare the values of test and control samples. *P*‐values of less than 0.05 were considered statistically significant.

## 3 RESULTS

### 3.1 Clinical characterization and novel allelic variants of individuals with MFS/Marfanoid disease

### 3.2 Subject XH253

**Figure 1:**
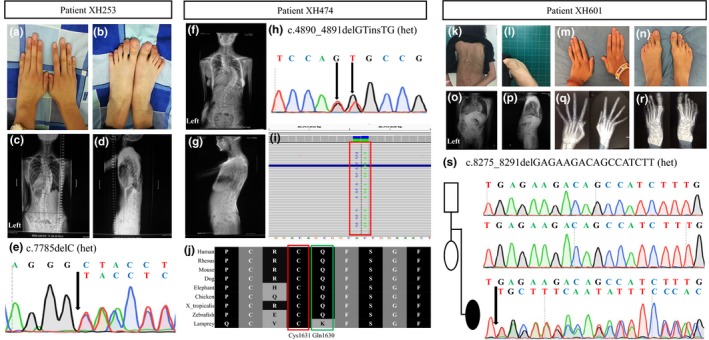
Clinical and genetic manifestations of the research subjects. (a, b) Pictures of Subject XH253 indicated bilateral arachnodactyly of hands and feet. (c, d) The anterior and lateral view of XH253 demonstrated scoliosis on whole spine X‐ray images. (e) Sanger sequencing of XH253 verified a novel heterozygous frameshift variant c.7785delC (p.Tyr2596Thrfs*86) affecting *FBN1*. (f, g) The anterior and lateral view of Subject XH474 demonstrated scoliosis on whole spine X‐ray images. (h) Sanger sequencing verified monoallelic missense mutations c.4890_4891delGTinsTG (p.Gln1630_Cys1631delinsHisGly) affecting *FBN1*. (i) Integrative Genomics Viewer (IGV) displays varying level of alignment reads detail depending on the zoom level and uses red box and transparency to highlight monoallelic missense variants in the exome data from XH474. (j) Conservation analysis of amino acid residues of *FBN1* among vertebrates. Lines indicate homologous amino acid sequences in selected vertebrates. Note the strong conservation of Cys1631 among vertebrates highlighted in red and Gln1630 highlighted in green, respectively. (k, l, m, n) Photographic images of Subject XH601 who presented with scoliosis, short stature and subcutaneous fat reduction and arachnodactyly anomalies of hands and feet. (o, p) The anterior and lateral view demonstrated severe scoliosis on whole spine X‐ray images. (q, r) Hand and foot radiographs showed bilateral metacarpophalangeal dislocation, interosseous atrophy, claw hands, and dolichostenomelia. (s) Sanger sequencing of XH601 parent–offspring trio verified a novel de novo heterozygous frameshift mutation c.8275_8291delGAGAAGACAGCCATCTT (p.Glu2759Cysfs*9) affecting *FBN1*.

The proband was a 10‐year‐old Chinese girl (Figure 1a‐d) who presented with kyphoscoliosis confirmed by radiologic imaging. She was referred to our orthopedic spine specialist for further evaluation and management. Whole spine X‐ray examination revealed scoliosis with three curves. Cobb angle of the major curvature was 43°, and she displayed a flat back and thoracolumbar kyphosis. Transthoracic echocardiographic evaluation revealed moderate mitral valve insufficiency and an aortic root measurement of 3.6 cm (Z‐score = 4.5 when standardized to age and body surface area). Skeletal system abnormalities such as long and thin limbs, and arachnodactyly were observed in the proband. Diminution of vision (myopia) occurred when she was 5 years old. The proband fulfilled the clinical criteria for classical MFS according to Ghent classification (Table 1), with a total of 7 points for the score in the revised Ghent criteria (Loeys et al., 2010). A novel heterozygous frameshift deletion, c.7785delC (c.7785del, p.Tyr2596Thrfs*86), was identified in *FBN1* in the proband via targeted NGS, and subsequently confirmed by further validation using an orthogonal experimental approach of Sanger sequencing (Figure 1e).

**Table 1:** Clinical features of Marfan syndrome patients reevaluated according to the revised Ghent nosology

Subject		XH253	XH474	XH601
	General patient information			
	Gender	F	F	F
	Age (years)	10	16	9
	Height (m)	1.72	1.71	1.36
	Weight (kg)	48	48	20.5
	Major Cobb angle (degrees)	43	85	117
	Major Curve type	R‐T	R‐T	R‐T
	Curve number	3	3	2
	Curve details	UTC (38), MTC (43), LC (16)	UTC (60), MTC (85), LC (40)	MTC (117), LC (89)
	Treatment	Surgery	Surgery	Surgery
Causal *FBN1* variant				
	Nucleotide alteration	c.7785delC	c.4890_4891delGTinsTG	c.8275A8291delGAGAAGACAGCCATCTT
	Protein change	p.Tyr2596Thrfs*86	p.Gln1630_Cys1631delinsHisGly	p. Glu2759Cysfs*9
	Variant reported in MFS	None	p. Cys1631Gly	None
MFS evaluation				
	Diagnosis	MFS	MASS	Marfanoid
	Aortic root dilation (z‐score)	4.5	1.3	1.6
	*Ectopia lentis*	A	A	A
	Systemic features score	7/20	8/20	7/20
	Wrist/thumb sign	P	P	A
	Pectus carinatum/ excavatum	A	A	A
	Hindfoot deformity	A	A	A
	Pneumothorax	A	A	A
	Dural ectasia	A	A	A
	Protrusio acetabuli	A	A	A
	Upper/lower segment	0.92	0.94	0.86
	Scoliosis or thoracolumbar kyphosis	P	P	P
	Reduced elbow extension	NA	NA	NA
	Facial features (3/5)	P	P	P
	Skin striae	A	A	A
	Myopia	−0.7 dioptries	NA	−0.4 dioptries
	Mitral valve prolapses (all types)	P	P	P

Abbreviations: A, absent; F, female; LC, lumbar curve; MASS, myopia, mitral valve prolapses, borderline (Z < 2) aortic root dilatation, striae, skeletal findings phenotype; MFS, Marfan syndrome; MTC, major thoracic curve; NA, not available; P, present; R‐T, right thoracic spine; UTC, upper thoracic curve.

### 3.3 Subject XH474

The proband was a 16‐year‐old Chinese girl (Figure 1f,g) who presented with *pectus excavatum* and scoliosis confirmed by radiologic imaging. Transthoracic echocardiographic evaluation revealed anterior mitral leaflet prolapse and moderate mitral valve insufficiency and an aortic root diameter measurement at the level of the sinuses of Valsalva of 2.6 cm (Z‐score = 1.3 when standardized to age and body surface area). Skeletal system abnormalities were present, such as tall stature with a height of 171 cm (P_97_; +3SD), slender body habitus with a weight of 48 kg (P_25_, −1SD), and very thin and long upper and lower limbs. Both of her hands indicated an appearance of arachnodactyly; wrist sign and thumb sign were positive. An *FBN1* mutation is identified in this sporadic case but aortic root measurements are still below Z‐score = 3 (Z = 1.3), thus according to the revised 2010 Ghent nosology, the term “potential MFS” should be used until the aorta reaches threshold size. Herein, the subject here should be categorized as “potential MFS” (Table 1), with a total of 8 points for the systemic score according to the revised Ghent criteria (Loeys et al., 2010). Heterozygous variants, c.4890_4891delGTinsTG (p.Gln1630_Cys1631delinsHisGly) in *FBN1*, were identified in the proband via targeted NGS and validated by Sanger sequencing (Figure 1h). The variants were confirmed to be *in cis* and comprising a complex allele resulting from a dinucleotide variant by visualizing read alignment views from the Integrative Genomics Viewer (IGV) (Figure 1i). Notably, the missense variant of c.4891T > G (p.Cys1631Gly) has been previously reported as a disease‐causing mutation in a classical MFS individual, whose clinical course and phenotype were characterized by aortic root dissection, aortic root dilation, mitral valve regurgitation, tricuspid valve prolapse, *ectopia lentis* (Arbustini et al., 2005). Conservation analysis of amino acid residues of Gln1630 and Cys1631 in *FBN1* demonstrates that they are highly conserved throughout evolution and across many selected species, suggesting they are required for the normal function of the protein. Thus, the monoallelic missense variants were most likely damaging and putatively deleterious and possibly accounted for “potential MFS” in this proband (Figure 1j).

### 3.4 Subject XH601

The proband was a 9‐year‐old Chinese girl (Figure 1k,l). When she was 4 years old at the time of her first evaluation, she presented with a height of 101 cm (P_25_; −1SD), an extremely thin body habitus with a weight of 12 kg (P_3_, −3SD). Physical examination revealed bilateral arachnodactyly (Figure 1m,n), uneven asymmetric back while bending and congenital dislocation of the hip joint. Whole spine X‐ray examination displayed severe right thoracic scoliosis with a main Cobb angle of 117° (Figure 1o,p). Transthoracic echocardiogram demonstrated anterior and posterior mitral leaflet prolapse and moderate mitral valve insufficiency without apparent aortic diameter enlargement at the sinuses of Valsalva or aortic root dissection. Ocular system abnormalities indicated bilateral downslanting palpebral fissures, epicanthus, and astigmatism. However, there were no signs or symptoms of *ectopia lentis*. The predominant features in this subject were an extreme congenital lack of subcutaneous fat, consistent with a lipodystrophy by physical exam, and a subsequent bodily progeroid appearance. However, this proband did not meet the revised Ghent criteria for classical MFS. Consecutive follow‐ups and assessment of the proband were conducted. Upon genotype‐driven reverse phenotyping of the proband when she was 9, the proportion of upper and lower segment lengths was basically normal with a ratio of 0.87. The proband had a height of 136 cm (P_50_ ,0). For comparison, her father's height measurement was 170 cm and her mother's was 160 cm. The body mass index (BMI) of the proband was extremely low (11.1 kg/m^2^) potentially due to disproportionate weight gain by age. More specifically, the subject had an extremely thin habitus with a weight of 20.5 Kg (P_3_, −3SD). Furthermore, hand and foot radiographs to potentially assess “bone age” suggested bilateral metacarpophalangeal joint dislocation, interosseous atrophy and dolichostenomelia (Figure 1q,r). The proband was eventually diagnosed with Marfanoid–progeroid–lipodystrophy syndrome (Table 2). A novel heterozygous frameshift deletion, c.8275_8291delGAGAAGACAGCCATCTT (c.8275_8291del; p.Glu2759Cysfs*9), was identified in the proband via targeted NGS. We subsequently confirmed this variant in Subject XH601, and it arose de novo through parental Sanger sequencing and trio analysis (Figure 1s). In addition to Sanger sequencing validation, we also performed in silico simulation of protein product using GENETYX (Ver.18, GENETYX CORPORATION; https://www.genetyx.co.jp), which also showed that this deletion caused a frameshift variation in the open reading frame (ORF). The resultant premature termination codon (PTC) will lead to a truncation of FBN1 protein to 2,767 amino acids (p.Glu2759Cysfs*9) (Supplementary Figure S1).

**Table 2:** Clinical manifestations of previously reported and our patient XH601 with Marfanoid–progeroid–lipodystrophy (MPL) syndrome with allelic truncating mutations in *FBN1*

	Graul‐Neumann et al. (2010)	Goldblatt et al. (2011)	Horn and Robinson et al. (2011)	Takenouchi et al. (2013)	Jacquinet et al. (2014)	Garg and Xing et al. (2014)		XH601 (this study)
						Patient 1	Patient 2	
Clinical characteristics								
Age (year)	27	20	3.5	10	16	23	17	9
Gender	Female	Male	Female	Female	Female	Female	Female	Female
Ancestry	Caucasian	Caucasian	Caucasian	Asian (Japanese)	Caucasian	Hispanic	Caucasian	Asian (Chinese)
Height (cm; [centile])	170	50–75	108	149	177	157.5	176	136
Weight (Kg; [centile])	39	<3	14.5	21.7	41.8	26.4	41.9	20.5
Causal *FBN1* mutation								
cDNA analysis	c.8155_8156del	c.8156_8175del	c.8226+1G>T, splice mutation, exon 65 skipping	c.8175_8182del	c.8226+1G>A, splice mutation, exon 65 skipping and subsequent frameshift	c.8206_8027insA	c.8226+1G>T, splice mutation, exon 65 skipping	c.8275_8291del
Protein change	p.(Lys2719Aspfs*18)	p.(Lys2719Thrfs*12)	p.(Glu2742Glufs*43)	p.(Arg2726Glufs*9)	p.(His2685Ilefs*9)	p.(Thr2736Asnfs*1)	p.(Glu2742Glufs*43)	p.(Glu2759Cysfs*9)
Adjacent to Exon 65 in *FBN1*	Y	Y	Y	Y	Y	Y	Y	Y
Inheritance pattern	De novo	De novo	De novo	De novo	De novo	De novo	De novo	De novo
BMI (kg/m^2^)	13.3	NA	12.4	9.8	13.3	10.6	13.5	11.1
Body fat (%)	20.5	NA	NA	NA	NA	28.4	27.7	NA
Premature birth	Y	Y	Y	Y	Y	Y	Y	Y
Birth weight (Kg)	1.78	1.04 (<3)	1	1.427	1.72	1.19	1.17	NA
Birth length (cm)	41.5	NA	40	40	45	40	40.75	NA
Progeroid appearance	Y	Y	Y	Y	Y	Y	Y	Y
Subcutaneous fat reduction	Y	Y	Y	Y	Y	Y	Y	Y
Arm span/height	0.99	NA	NA	NA	NA	0.94	0.98	NA
Upper/lower segment	0,95	NA	NA	NA	NA	0.99	0.8	0.87
Proptosis	Y	Y	Y	Y	Y	Y	Y	Y
DPF	NA	NA	NA	NA	NA	NA	NA	Y
Myopia	Y	Y	*N*	Y	Severe	Y	Y	Y
*Ectopia lentis*	Bilateral	Bilateral	*N*	*N*	*N*	Left eye	*N*	*N*
High‐arched palate	Y	Y	Y	NA	NA	Y	*N*	NA
Pectus excavatum	NA	Y	NA	Y	*N*	*N*	Y	*N*
scoliosis/ kyphosis	Kyphosis	NA	NA	NA	*N*	Scoliosis	Scoliosis	Severe scoliosis
Cobb angle (degrees)	NA	NA	NA	NA	NA	NA	NA	117
Wrist sign	Y	Y	NA	NA	NA	Y	Y	Y
Thumb sign	Y	*N*	NA	NA	NA	Y	Y	Y
BMJD	NA	NA	NA	NA	NA	NA	NA	Y
Interosseous atrophy	NA	NA	NA	NA	NA	NA	NA	Y
Arachnodactyly	NA	NA	NA	Y	Y	NA	NA	Y
Hyperextensible digits	NA	Y	NA	Y	Y	Y	Y	Y
Pes planus/valgus	NA	Pes planus	NA	NA	Pes valgus	NA	Pes planus	NA
Easy bruisability	Y	Y	NA	NA	NA	*N*	*N*	Y
MVPS	Y	*N*	*N*	*N*	*N*	NA	Y	Y
Arrested hydrocephalus	NA	Y	Y	Y	NA	NA	NA	NA
RGC	NA	NA	NA	Hydronephrosis	*N*	NA	NA	*N*
Dural ectasia	Lumbosacral	NA	NA	Y	Y	NA	NA	*N*
Hypertension	NA	NA	NA	Y	NA	NA	NA	*N*
CHD	NA	NA	NA	NA	NA	NA	NA	Y

Abbreviations: BMJD, bilateral metacarpophalangeal joint dislocation; CHD, congenital hip dislocation; DPF, downslanting palpebral fissures; MVPS, mitral valve prolapse syndrome; *N*, no; NA, not available; RGC, renal/genitourinary complications; Y, yes.

Moreover, these two allelic truncating variants, p.Tyr2596Thrfs*86 and p.Glu2759Cysfs*9, were not present in ExAC, 1,000 Genomes, ESP6500, gnomAD, the Universal Mutation Database for *FBN1* (UMD‐FBN1; http://www.umd.be/FBN1/) (Collod‐Beroud et al., 2003) and our in‐house control dataset. Clinical and genetic characteristics of subject XH253 with classical MFS and XH474 with “potential MFS” based on the diagnostic Ghent criteria (Loeys et al., 2010) are presented in Table 1.

### 3.5 NMD degradation prediction

**Figure 2:**
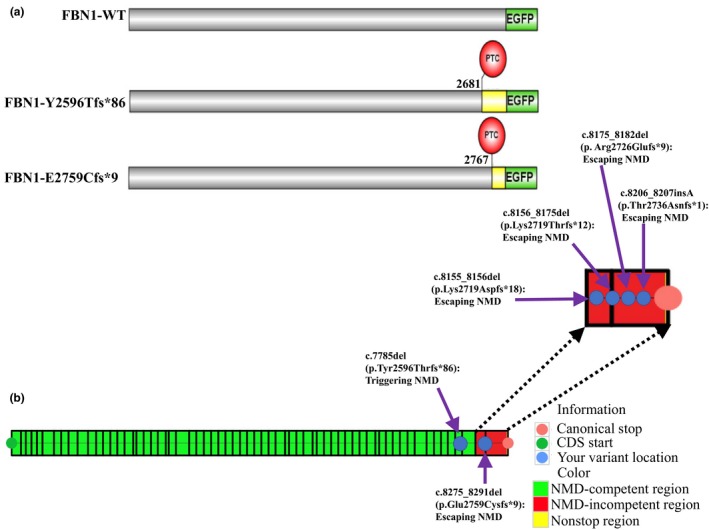
Schematic representations and NMD predictions of the p.Y2596Tfs*86 and p.E2759Cfs*9 mutations other MPLS‐affected frameshift mutations in *FBN1*. (a) Schematic diagrams of full‐length WT and mutant EGFP‐FBN1 plasmids transiently expressed in HEK293T. The numbers indicate the amino acid positions in *FBN1*. Locations of protein truncation caused by PTC are marked with red circles, respectively. The resulting frameshift and reduction in the deduced amino acid sequence caused by the p.Y2596Tfs*86 and p.E2759Cfs*9 mutations are indicated by the yellow region, respectively. (b) Prediction of frameshift variants that are potentially subject to NMD escape or NMD degradation. The mutation of p.Y2596Tfs*86 in *FBN1* is predicted to be subject to degradation by triggering NMD. The p.E2759Cfs*9 mutation in *FBN1* is predicted to escape NMD. All of other MPLS‐affected frameshift mutations in *FBN1* are predicted to escape NMD.

The variant of Y2596Tfs*86 identified in XH253 was a single nucleotide frameshift deletion leading to a PTC in exon 64, while E2759Cfs*9 identified in XH601 was a frameshift deletion leading to a PTC in exon 66, the final exon of *FBN1* (Figure 2a). Although both variants are very close in linear space, they may lead to distinct alterations to the *FBN1* transcript. To test this hypothesis, we performed NMD predictions of the two truncating variants on NMDescPredictor (Coban‐Akdemir et al., 2018). We predicted that the transcripts with Y2596Tfs*86 would go through NMD (NMD‐competent), and that the E2759Cfs*9 transcripts would escape NMD, that is, NMD‐incompetent (Figure 2b). Using the same analytical tools, we further tested whether previously reported protein‐truncating *FBN1* frameshift variants implicated in MPLS subjects (Garg & Xing, 2014; Goldblatt et al., 2011; Graul‐Neumann et al., 2010; Takenouchi et al., 2013) uniformly shared the mechanism of escaping NMD. Our analysis shows that all of the truncating *FBN1* frameshift variants within the C‐terminal gene region may escape NMD, supporting the contention that the underlying disease mechanism in MPLS is the same and distinct from MFS (Figure 2b). These findings implicate a mechanism distinct from loss‐of‐function (LoF) alleles, and potentially gain‐of‐function (GoF) variant alleles (Coban‐Akdemir et al., 2018), at the *FBN1* locus causing MPLS.

### 3.6 Perturbation of native aggregation process by MPLS‐causative truncating mutation

**Figure 3:**
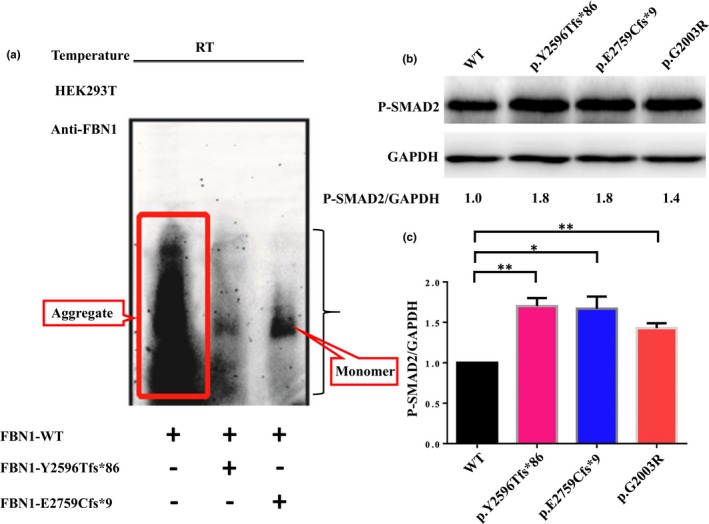
Functional effects of the Y2596Tfs*86 and E2759Cfs*9 mutations on *FBN1* protein expression. (a) Detection of SDS‐resistant aggregates by SDD‐AGE in cell lysates of HEK293T transiently expressing EGFP‐FBN1 fusions were investigated by SDD‐AGE and western blot. Expression of the proteins was induced for 48 hr and detected with a monoclonal FBN1‐specific antibody. (b) Elevated phosphorylation of SMAD2 (pSMAD2) in individuals with truncating *FBN1* variants, respectively. G2003R is used as a positive control. GAPDH is used as a loading control. pSMAD2/GAPDH ratio is shown to be normalized to the unaffected control (WT). (c) Grayscale analysis results show significantly upregulated (pSMAD2) in constructs encoding truncating *FBN1* variants. EV denotes empty vector (pEGFP). RT: room temperature. Data are represented as mean ± *SD* of three independent experiments. * denotes *p* value < .05 and ** denotes *p* value < .01.

To further determine if the mutant FBN1 protein in MPLS is able to lead to dominant‐negative or GoF effects, plasmids expressing mutants (pEGFP‐FBN1‐Tyr2596Thrfs*86; pEGFP‐FBN1‐Glu2759Cysfs*9) and WT plasmid (pEGFP‐FBN1) were co‐transfected into HEK293T cells at a 1:1 ratio. After 48 hr of expression, lysates from cells expressing EGFP‐FBN1 fusions were analyzed by SDD‐AGE. Detection of SDS‐resistant aggregates by SDD‐AGE in cell lysates of HEK293T transiently expressing EGFP‐FBN1 fusions were investigated with SDD‐AGE and western blot. Protein expression was induced for 48 hr and detected with a monoclonal FBN1‐specific antibody. Remarkably, WT plasmid (EGFP‐FBN1 fusions) had the SDS‐resistant properties of an amyloidogenic protein structure. Since amyloid formation is time‐ and concentration‐dependent, we set the time of transient expression to 48 hr. WT plasmid (EGFP‐FBN1 fusions) aggregated after 48 hr of expression, whereas co‐transfection of pEGFP‐FBN1‐Glu2759Cysfs*9 and WT plasmid resulted in the presence of a small fraction of monomer protein. This suggests that pEGFP‐FBN1‐Glu2759Cysfs*9 apparently prohibited the native aggregation process of WT plasmid, possibly revealing an intracellular dominant‐negative mechanism. In addition, we observed that co‐transfection of pEGFP‐FBN1‐Tyr2596Thrfs*86 and WT plasmid presented a small fraction of aggregated protein. As this fraction was less than that of the WT plasmid, it can be suggested that Tyr2596Thrfs*86 may undergo an NMD mechanism thus leading to *FBN1* haploinsufficiency (Figure 3a). Moreover, these variations in the range of particle sizes were observed, and again were reproducible for individual EGFP‐FBN1 fusions validated by another two independent replication experiments.

### 3.7 Disruption of downstream TGF‐β signaling

Previous studies have demonstrated that deleterious *FBN1* mutations causing Marfan syndrome result in upregulated endogenous transforming growth factor β (TGF‐β) receptor signaling (Andelfinger, Loeys, & Dietz, 2016). Such signaling can be measured in plasma or indirectly measured through aberrant activation of downstream targets, including excessive phosphorylation of SMAD2 (pSMAD2) (Verstraeten, Alaerts, Van Laer, & Loeys, 2016). To determine the functional consequences of the novel truncating *FBN1* variant of E2759Cfs*9 in the MPLS subject, plasmids expressing mutant EGFP‐FBN1 cDNAs were transfected into HEK293T cells. Transfected cells with truncating *FBN1* variants of Y2596Tfs*86 (*p* = .007), E2759Cfs*9 (*p* = .017) and Gly2003Arg (*p* = .006) showed significantly elevated phosphorylation of SMAD2 (pSMAD2) in Western blots compared to the WT plasmid (Figure 3b,c). This observation is consistent with the contention that the TGF‐β signaling pathway is perturbed in a SMAD‐dependent manner in all three subjects.

### 3.8 Genotypic and phenotypic features of reported MPLS subjects

Intriguingly, the mechanism underlying *FBN1*‐related diseases involves either loss‐of‐function (LoF) or NMD surveillance pathway escape as conveyed by allelic truncating mutations. Such distinct mechanisms could contribute to distinct disease phenotypes of varying severity. According to a prior report, *FBN1* LOF mutations led to classical MFS (Park et al., 2017), while predicted NMD surveillance pathway escape in *FBN1* can cause a MPLS phenotype (Romere et al., 2016). Subject XH601 in our cohort harbored a de novo variant of E2759Cfs*9 in *FBN1*, which is located in the final exon. Notably, our MPLS subject presented with bilateral downslanting palpebral fissures, epicanthus, and eye astigmatism. The main clinical features of this complicated disorder, clustered in Table 2, include accelerated aging and postnatal lipodystrophy, poor weight gain since birth, premature birth, hyperextensible digits and generalized subcutaneous fat reduction leading to a progeroid appearance of the body in all subjects. Mental and motor development remain mostly normal. Overlapping clinical features of MPLS with MFS are phenotypically diverse. Ocular system involvement like myopia has been observed in all individuals, although hyperextensible joints, arachnodactyly, and other significant signs of classical MFS are not always present, that is, mitral valve prolapse in 4 of 8, lumbosacral dural ectasia in 2 of 3 (5 data points unavailable), *pectus excavatum* in 3 of 8, and ectopia lentis in 3 of 8. Scoliosis was reported in two subjects aged 23 and 17 years, and kyphosis was reported in a subject aged 27 years, but not described in the others (Table 2). Remarkably, the major Cobb angles of Subject XH253 with classical MFS and XH474 with MASS were 43° and 85°, respectively. Our MPLS subject presented a thoracic scoliosis with a major Cobb angle magnitude of 117°–a condition much more severe compared to the MFS subjects.

## 4 DISCUSSION

In the present report, we provide direct evidence for potential dominant‐negative alleles in MPLS subjects caused by truncating *FBN1* variants escaping NMD. These genetic and functional investigations also include description of the first subject with MPLS of Chinese ancestry.

Several case reports previously identified an association between monogenic *FBN1* mutations and reduction of subcutaneous fat and/or progeroid features (Goldblatt et al., 2011; Graul‐Neumann et al., 2010; Jacquinet et al., 2014; Passarge et al., 2016; Takenouchi et al., 2013). Here, we have provided further evidence for the existence of a distinct genetic disease, MPLS, caused by *FBN1* mutations. The MPLS subject in our study is characterized by accelerated aging and postnatal lipodystrophy, disproportionate weight gain since birth, severe scoliosis, downslanting palpebral fissures, bilateral metacarpophalangeal joint dislocation, mitral valve prolapse, bilateral interosseous atrophy, and congenital dislocation of the hip. Intriguingly, this subject was previously diagnosed with Marfanoid disease (Table 1) due to an unclear molecular diagnosis. We consequently performed targeted NGS on the affected proband and her unaffected parents. As a result, we identified c.8275_8291del (p.Glu2759Cysfs*9) in *FBN1* as the responsible variant for the proband. Sanger sequencing was then conducted on genomic DNA from both parents to confirm the de novo occurrence and segregation with phenotypes. Subsequently, we proceeded with a comprehensive phenotypic analysis of all family members through intensive clinical follow‐up. By combining genetic data with reverse phenotyping, we eventually diagnosed the proband with MPLS. Notably, the severity of spinal deformity in the MPLS individual was significantly more severe than that in XH253 and XH474 (with classical MFS and potential MFS, respectively). We consider that MPLS is a distinct fibrillinopathy because it can be clinically distinguished from other fibrillinopathies, including classical MFS. To our knowledge, there are no other syndromes that simultaneously comprise the clinical phenotypes of marfanoid features, accelerated aging, postnatal lipodystrophy, poor weight gain since birth, and progeroid appearance. Particularly, the involvement of both reduced subcutaneous fat tissue and progeroid appearance is a remarkable characteristic of MPLS. These comprehensive findings predominate in determining the severity of defects in individuals and thus significantly influence the prognosis: lipodystrophic disorders are frequently associated with metabolic disturbance, such as insulin resistance and life‐threatening hypertriglyceridemia (Vantyghem et al., 2012). Such potential metabolic disturbances are particularly important to note prior to surgical intervention as has been noted for the ocular‐scoliotic form of Ehlers Danlos syndrome due to lysyl hydroxylase deficiency (Yeowell, Walker, Marshall, Murad, & Pinnell, 1995). Secondly, elucidation of the phenotype of MPLS has been limited due to the paucity of prior clinical reports, with only seven relevant subjects previously described. Up to now, it is known only to be caused by a single gene with a monogenic AD inheritance pattern for the disease trait. Finally, we have shown that, in terms of pathogenic mechanisms, the truncating *FBN1* mutations that cause MPLS cluster in 3’ gene regions encoding the extreme C‐terminal domains and these variant alleles represent a subset that differ from those that cause classical MFS. Most of such mutations will be anticipated to escape NMD and we propose these to function via a potential mechanism between GoF and dominant‐negative and not a LoF mechanism.

Fibrillin‐1 acts as the precursor to a recently described glucogenic hormone: asprosin (Davis et al., 2016a). Specifically, exon 65 of *FBN1* encodes 11 amino acids, and exon 66 of *FBN1* encodes 129 amino acids of asprosin (Romere et al., 2016). Recently, Chen et al. have successfully constructed a genetically modified rabbit model with a truncated C terminus of fibrillin‐1 involving the ultimate two exons which could recapitulate phenotypes associated with MPLS (Chen et al., 2018). Since individuals with low fibrillin‐1 level may fail to differentiate adipocytes to accumulate adipocyte lipids, truncating variants that occur affecting the ultimate two exons of *FBN1* are prone to cause lipodystrophic phenotypes (Davis et al., 2016b).

**Figure 4:**
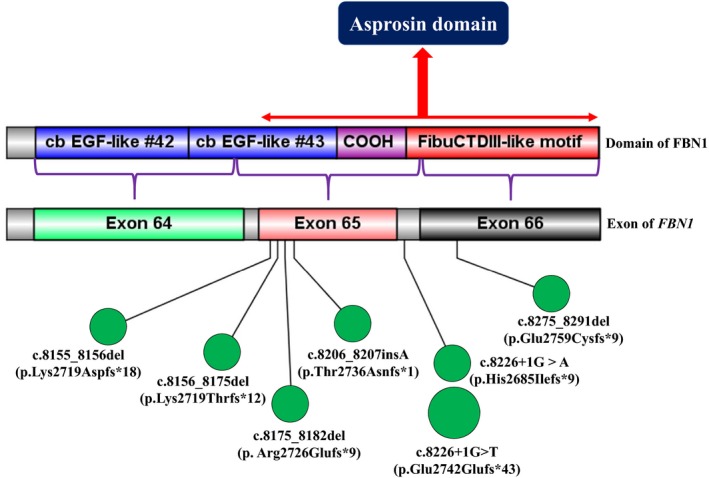
The partial gene structure of the C terminus of *FBN1* and overview of previously reported truncating variants in and around exon 65 in patients presenting with MPLS. Blue, purple, and red boxes denote specific domain. Light green, orange, and black boxes denote exons and the gray area between the two boxes denote introns. All truncating variants were annotated by small green circles except that c.8226+1G>T previously reported twice was annotated by a larger circle. COOH denotes COOH unique region. Deep blue box denotes purported asprosin encoded by the exons of *FBN1*.

PTCs located in the final coding exon are specifically prone to escape NMD and so are processed distinctly from those in internal exons regarding transcript degradation, leading to the stable translation of truncated proteins (Bayram et al., 2017). Excessive amounts of mutated protein are therefore produced (Inoue et al., 2004), leading to a dominant‐negative mechanism and the severe MPLS disease phenotype. Consistent with this interpretation, all truncating mutations associated with MPLS are located adjacent to the penultimate exon (exon 65) and are predicted to escape NMD (Figure 4). From this perspective, it is perhaps most parsimonious to postulate that the incorporation of relatively few mutant monomers would be sufficient to impair the systemic processes of microfibrillar assembly and function.

Aberrant activation of the TGF‐β pathway has been observed in MFS and may account for some of the musculoskeletal deformities, such as scoliosis (Buchan et al., 2014). The MPLS subject with a truncating *FBN1* variant presented with severe scoliosis, which suggests that the protein change also alters TGF‐β signaling. Our results showed that upregulation of the TGF‐β pathway in plasmids expressing mutant EGFP‐FBN1 cDNAs, confirming that the variant of E2759Cfs*9 identified in the MPLS subject has functional effects in a SMAD‐dependent manner in all three subjects.

Our study indicated that the two phenotypes associated with *FBN1* mutations, MPLS and MFS are caused by two distinct molecular mechanisms. The more complicated phenotype of MPLS, which had its first report in the Chinese population, is caused by a nonsense mutation within the glucogenic hormone asprosin domain of *FBN1* (Romere et al., 2016). Truncated mutant FBN1 proteins were produced by the mutation. These proteins disrupt the process of native amyloid‐like structure conformation and exert potent intracellular dominant‐negative activity. In this view, abnormal protein derived from the mutant allele interacts and interferes with protein derived from the normal allele, consequently resulting in substantial loss‐of‐function. Supportive evidence includes (a) de novo monogenic AD inheritance pattern, and (b) aggregation of fibrillin‐1 in which the mutated monomer derived from the mutant allele impairs the fundamental structure of its wild‐type protein derived from the normal allele counterpart. This deficiency could be supported by SDD‐AGE analysis, suggesting that dominant‐negative interference is not restricted to enhanced proteolytic clearance of mutant microfibrils over time (Brenn, Aoyama, Francke, & Furthmayr, 1996), but rather could also occur at the level of intracellular aggregate formation processes. Consistently with our findings, Romere et al. have indirectly proved that some MPLS‐associated mutant *FBN1* alleles (c.8206_8207insA and 8226+1G>T) which are predicted to avoid mRNA NMD, have a dominant‐negative effect on secretion of asprosin from the corresponding WT alleles in both human and mouse (Romere et al., 2016). The more moderate phenotype, classical MFS, is caused by nonsense mutations that activate the NMD RNA surveillance pathway, thereby degrading mutant transcripts and resulting in *FBN1* haploinsufficiency. The data support the concept of distinct pathogenetic mechanisms, GoF versus LoF (Bayram et al., 2017; Coban‐Akdemir et al., 2018; Poli et al., 2018), for each well‐established subgroup of fibrillinopathy, which mostly hinges on the intrinsic features of fibrillin‐1 mutations. It is of crucial significance to dissect the locus heterogeneity, clinical relevance and overlapping clinical features with other disorders in a phenotype‐driven approach that characterizes much of clinical practice (White et al., 2018).

In conclusion, we provide direct evidence of dominant‐negative effects of truncating *FBN1* variants predicted to escape NMD in MPLS subjects. Our study expands the mutational spectrum of *FBN1* and highlights the potential molecular mechanism for MPLS subjects, which facilitates our understanding of genotype–phenotype correlations in *FBN1* to provide effective genetic counseling, implementation and timing of therapy (e.g. mitigation of TGF‐β hypersignaling, surgical intervention for cardiovascular complications or for scoliosis), or early intervention.

## CONFLICT OF INTEREST

J.R.L has stock ownership in 23andMe, is a paid consultant for Regeneron Pharmaceuticals, and is a co‐inventor on multiple the United States and European patents related to molecular diagnostics for inherited neuropathies, eye diseases, and bacterial genomic fingerprinting. The Department of Molecular and Human Genetics at Baylor College of Medicine derives revenue from the chromosomal microarray analysis and clinical exome sequencing offered in the Baylor Genetics Laboratory (http://bmgl.com).
