# Supplementary material for: Genetic and molecular mechanism for distinct clinical phenotypes conveyed by allelic truncating mutations implicated in FBN1
Source: Mol Genet Genomic Med. 2019 Nov 27;8(1):e1023. doi: 10.1002/mgg3.1023 (PMC6978264; doi:10.1002/mgg3.1023)
Supplement: Supplementary file 1 [file MGG3-8-e1023-s001.pptx]

## Slide 1
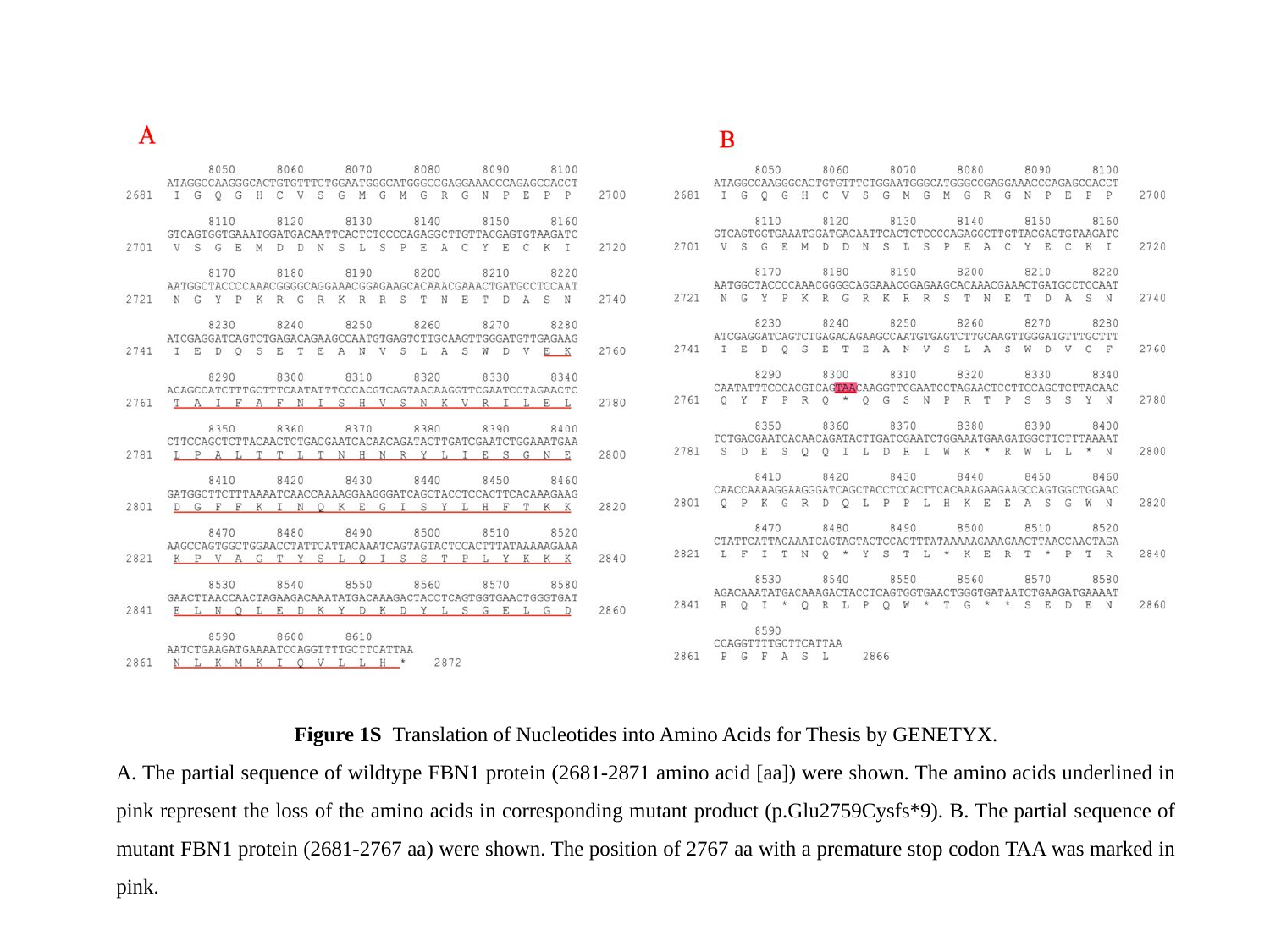

Figure 1S Translation of Nucleotides into Amino Acids for Thesis by GENETYX.
A. The partial sequence of wildtype FBN1 protein (2681-2871 amino acid [aa]) were shown. The amino acids underlined in pink represent the loss of the amino acids in corresponding mutant product (p.Glu2759Cysfs*9). B. The partial sequence of mutant FBN1 protein (2681-2767 aa) were shown. The position of 2767 aa with a premature stop codon TAA was marked in pink.
